# Probability of Self-Location in the Framework of the Many-Worlds Interpretation

**DOI:** 10.3390/e27040416

**Published:** 2025-04-11

**Authors:** Lev Vaidman

**Affiliations:** 1 School of Physics and Astronomy, Tel-Aviv University, Tel-Aviv 69978, Israel; vaidman@tauex.tau.ac.il; 2 Institute for Quantum Studies, Chapman University, Orange, CA 92866, USA

## Abstract

The growing interest in the concept of probability of self-location of a conscious agent has created multiple controversies. Considering David Albert’s setup in which he described his worries about consistency of the concept, I identify the sources of these controversies and argue that defining “self” in an operational way provides a satisfactory meaning for the probability of self-location of an agent in a quantum world. It keeps the nontrivial feature of having subjective ignorance of self-location without ignorance about the state of the universe. It also allows defining the Born rule in the many-worlds interpretation of quantum mechanics and proving it from some natural assumptions.

## 1. Introduction

I am very pleased to contribute to this book celebrating 100 years of Born’s rule. I am an academic great-great-grandchild of Max Born. My Ph.D. advisor Yakir Aharonov obtained his doctorate under David Bohm who was the student of Robert Oppenheimer, the student of Max Born.

I do not know what role Born’s rule played in the research of Robert Oppenheimer, although it is clear that it is needed for calculating the probability of a nuclear chain reaction. David Bohm [1] took the Born rule as a postulate in his theory of hidden variables. It allowed him to remove randomness from dynamical evolution by putting it into the initial distribution of Bohmian particle positions in the universe. Yakir Aharonov also adopted the Born rule (less explicitly) with another proposal to avoid dynamical randomness [2]. He postulated a backward evolving quantum state which corresponds to the outcomes of future measurements which exhibit Born rule statistics. Aharonov, Bergmann, and Lebowitz [3] generalized the Born rule to measurements performed on pre- and post-selected quantum systems (in contrast to measurements on systems which are pre-selected only)—what is known as the ABL rule. The time-symmetric approach to quantum measurements created significant controversy [4,5], and my first contribution was the defense of the ABL rule [6,7]. Since then, the Born rule has played a central role in my research, including recent analysis of the derivations of Born’s rule [8].

Today, we accept randomness in nature and it is Born’s rule that is responsible for this [9]. However, I remained with the conservative view preferring deterministic theories, and I consider that my most important contribution related to Born’s rule is finding a way to define its counterpart in a deterministic quantum theory, the many-worlds interpretation (MWI) [10]. Tappenden [11] named it the Born–Vaidman rule. I introduced this concept through discussion of the probability of self-location which has recently gained increasing interest. The Stanford Encyclopedia of Philosophy assigns a specific entry to self-locating beliefs [12]. Very recently, several authors have seriously considered speculations according to which we live in computer simulations [13,14], although Adlam [15] is skeptical regarding the implications of these applications of self-locating probabilities in scientific contexts. Perhaps the biggest surprise is an enormous interest in the Elga–Lewis controversy about the Sleeping Beauty scenario [16,17], as well as significant attention to application of this concept to defeat Dr. Evil [18].

According to the MWI, after performing a quantum measurement, an observer splits into agents in different worlds, and one may want to attach probabilities to the agent of being in different worlds. The situation of self-locating in the MWI is closer to the case of Dr. Evil considering the probability of being Dr. Evil on the moon or being Dup on Earth (see [18]) than to the case of Sleeping Beauty being uncertain about the day she is currently in (see [16]). Note that there is also an extensive literature on Sleeping Beauty in the quantum world [11,19,20,21,22,23,24,25,26].

In this paper, I will defend the concept of self-location probability in the MWI framework. MWI was mainly introduced to solve the measurement problem of quantum theory. Arguably, the most serious difficulty of the MWI is understanding the probability of the outcomes of quantum experiments. MWI is a deterministic theory, but this alone does not preclude the concept of probability. When we go to the casino, we believe that the outcome of roulette is a unique function of the precise details of the roll, but we view it as random because of our ignorance of these details. In quantum experiment, however, there is nothing relevant which we are ignorant about prior to the experiment. All possible outcomes take place, so it seems that there is no coherent way to talk about probability for a particular outcome. I will argue that the concept of self-location resolves this problem, and thus it is necessary for understanding the Born statistics of the results of quantum experiments in the framework of the MWI. In my view, numerous controversies about this concept can be satisfactorily resolved by a precise definition based on the operational meaning of the identity of an agent. For my analysis, I will consider a teleportation scenario introduced by Albert [27]. I disagree with Albert about some (important) details, but I agree with his conclusions that self-location probability in the MWI requires a radical modification of the scientific paradigm. However, contrary to Albert, I will argue that this is the best way to solve the measurement problem of quantum mechanics.

I will start with presenting Albert’s classical teleportation setup in Section 2 and the quantum setup in Section 3. In Section 4, I will argue that the MWI without ontology beyond the quantum state of the universe does not allow a popular four-dimensional worm view of an agent. In Section 5, I will modify Albert’s quantum setup to provide a meaningful concept of self-location probability and will argue that although it is radically different from the standard scientific paradigm of analysis of nature in objective terms, we do not have a better alternative. After establishing the meaning of self-location probability in subjective terms, in Section 6, I turn to the question of a quantitative description of this probability. What are the assumptions, if any, to derive the Born rule? In particular, I will show how it can be derived from assuming that local unitary operation cannot affect anything in remote locations. In Section 7, I briefly summarize my optimistic view on self-location probability in the MWI.

## 2. Albert’s Setup: Classical Teleportation

To set up the stage for my analysis, I will use a setup considered by Albert [27]:

Captain Kirk is about to step into the transporter, to beam down to the planet below. He happens to know that the transporter is malfunctioning at the moment—to wit: the transporter is going to make two Kirks on the surface of the planet out of the one that steps in on the ship, each of them dressed in a different color—one blue, one green. Both the Kirks initially arrive on the planet with their eyes closed—and (more generally) with no indication whatever of which particular one of the two Kirks on the planet they are. But each of them knows that they have arrived on the planet, and each one says to himself, correctly, that “there is now some perfectly determinate fact of the matter about which particular one of those two Kirks I am”. And each of them wonders which particular one they might be. And then they open their eyes and find out. So, consider this moment, after they have arrived on the planet but before they have opened their eyes, when each of the Kirks is wondering which particular one of the Kirks he is. Lots of people—a whole academic industry of people—seem to think it makes sense for these Kirks to talk to themselves about “the probability that, when I open my eyes, I will find that I am this particular Kirk or that particular Kirk”, or “the probability that, when I open my eyes, I will see that I am wearing a blue outfit”. And this kind of talk would seem to amount to a way out of the puzzle that I mentioned above—this would seem to offer us a way of talking intelligibly about ‘the probability that I am about to have this experience’ and ‘the probability that I am about to have that experience’ even in circumstances in which I already know absolutely everything about the future physical condition of the world. The idea is that even in circumstances in which I have no ignorance whatsoever about the future objective physical condition of the world, I might still be ignorant—just as these various Kirks are ignorant, when they are standing on the surface of the planet with their eyes closed—about where I am located in it. People call these sorts of probabilities “self-locating” probabilities.

There is nothing quantum in this setup, but even in this situation, Albert finds discussion of probability confusing. I am interested in the quantum case, but let me first offer a simple operational analysis of this classical case to prepare the ground for the quantum setup.

Most analyses of self-location beliefs introduce “worlds” and “centered worlds” [28]. Instead, I propose to focus on the identity of the agent, arguing that this move will solve the problem. In Albert’s setup, we have two “Kirks” on the planet. They differ by their outfit: green or blue, but since we are talking about two people in the framework of classical physics, they also must differ in their location on the planet. Thus, we have one Kirk, say, with a green outfit on the left and another Kirk with a blue outfit on the right. Tappenden [29] considered a “single-mind” Kirk, defined by his sentient state (which can be considered as the relative state of particles in Kirk’s brain) irrespective of the location in space relative to the planet. This “Kirk” is present both on the left and on the right. I will argue below that this metaphysical approach to identity of an agent will not lead to an operational concept of probability.

The operational meaning of the subjective probability of a particular fact for an agent can be modeled as the part of the dollar that the agent is ready to pay for a game in which he gets a dollar, if the fact is true (see [30]). Since all Kirks have the same sentient state, they must have identical answers to every question. If an external agent asks one of the Kirks to bet on being the Kirk with a blue outfit, will he be ready to pay fifty cents for the game? For Albert’s setup, we expect probability 0.5.

Let us describe the situation with more care. An external agent comes close to one of the Kirks, who is awake but did not open his eyes, and offers this game for fifty cents: Should Kirk agree to play? Albert, I believe, would say that Kirk has no clue about the color of his outfit and that he has to refuse. I agree that Kirk should refuse. The external agent sees the color of Kirk’s outfit and would not offer the game to Kirk in a blue outfit. Thus, Kirk is certain to lose. However, I think that Kirk on the left and Kirk on the right have a subjective probability 0.5 for the green (as well as blue) outfit. When an external agent approaches, each of the Kirks is ready to pay fifty cents for a game in which they get a dollar when the color of their outfit is the one *they* chose (before opening their eyes). All that is required to have an expected payoff is that Kirk has the ability to choose, keeping external agents ignorant about his choice. This ability will not help to define the probability for a “single-mind” Kirk because there is no matter of fact about the color of his outfit.

## 3. Albert’s Setup: Quantum Teleportation

Let us move on to the quantum case. I name it “quantum teleportation” since we have a scenario with a transporter and quantum superposition, but note that it is very different from what is usually named quantum teleportation: the protocol that transfers a quantum state using prior entanglement and a classical channel. Albert continues:

Imagine (then) that the adventures of Kirk in the transporter correspond to an actual, quantum-mechanical, Everettian splitting. Imagine (that is) a quantum-mechanical measurement—say a measurement of the x-spin of an electron that is initially in an eigenstate of z-spin—whose result is encoded in the color of Kirk’s outfit. Kirk’s outfit is like the pointer on the measuring-device, the color of his outfit is the position of the pointer, and Kirk himself is the sentient observer—and he becomes aware of the outcome of the experiment when he opens his eyes. And the thought is that the quantum-mechanical probability that Kirk will see this or that particular outcome of this measurement is precisely the self-locating probability that “I, Kirk” or “I, among the Kirks”, or whatever it is that I call myself, am going to find, on opening my eyes, that my outfit is this or that particular color.

In classical teleportation, we had two Kirks on the planet: one on the left and one on the right. We do not have two Kirks in the quantum case. I think the option that Albert considers: “I, among the Kirks” does not exist. We consider Kirk before he opens his eyes when he is not aware of the outcome. My reading of Albert is that whatever Kirk is, at this stage, there is no entanglement between Kirk and his outfit, so there are no two different states of the captain. There is a single Kirk with an outfit in a superposition of being blue and green.

One may be concerned about the issue of decoherence: How can the outfit be in a superposition of macroscopically distinguishable states (green and blue), while Kirk, wearing it, is in a pure state not entangled with the states of the outfit? A model in which the outfits with different colors cause difference in the Kirk’s brain only after he opens his eyes seems possible (surely possible if teleportation of Kirks is possible), and this is the scenario considered here.

Note that even if we consider a scenario in which decoherence will lead to a macroscopic number of molecules in Kirk’s brain to be strongly entangled with the color of the outfit, I still would prefer not to consider two Kirk’s here. “Kirk” is a quantum state of particles which specifies a three-dimensional pattern in a shape of the captain (see [31]). The superposition of such states in remote locations corresponds to multiple Kirks. The superposition of states in the same macroscopic location, but describing brains in different knowledge states of the color of the outfit also corresponds to different Kirks. However, in the situation described in Albert’s setup, I see only one Kirk. He knows the situation: the outfit might be entangled with parts of his brain, but not the parts of the brain that supervene on his awareness now. Thus, for Kirk with awareness, there is no matter of fact about the definite color of the outfit. Kirk knows that the outfit and parts of his brain are in mixed states. In the semantics of [32], Kirk is still in the state of “absent self-location uncertainty”. There are no two Kirk’s because there are no two different macroscopic bodies corresponding to two different sentient states. Given decoherence, there are two different autonomous branches which do not exhibit interference, but there is no “Kirk” who is uncertain in which branch he is.

Albert is not the only one who takes this (in my view, illegitimate) approach. Tittelbaum [33] writes in Section 10:

… For instance, take the time immediately after the [Stern–Gerlach] experiment has been run but before anyone has observed its outcome. At that time, there are two agents in two universes. Each of those agents is about to measure a different outcome, …

I deny the possibility of talking about two agents at this stage.

More recently, Wilson [34] writes:

… subsequent to a Stern–Gerlach measurement I can know that there is an x-spin-up world and a x-spin-down world but not know which of these two worlds I am in. These self-locating contents are no mere curiosity: for many Everettians they provide the subject matter for objective probabilities in EQM [35,36,37,38,39].

I argue that in Wilson’s setup, I am in both worlds together, so I cannot ask myself in which world am I. In the literature mentioned by Wilson, there are formal semantic definitions for this question, but I argue that the consideration of this question is improper. For example, the derivation of the Born rule by Sebens and Caroll [39] fails if the question lacks operational meaning for alternatives (see [32,40] and other discussions of the Sebens–Caroll proof [41,42]).

The operational meaning discussed here is the possibility (even if it is a gedanken possibility with today’s technology) using physical (local) interactions to observe the difference between the agents (Kirks) before they open their eyes (i.e., during a short period of time and not by viewing the agent’s history). The “agent” is considered a minimal part of the brain that contains information about its sentient state. The “brain” is modeled as a wave function of relative spatial coordinates of its constituents together with the position of the brain relative to a macroscopic frame of reference. The absolute position should be considered since macroscopic objects (agents) cannot, by definition, be in a superposition of different locations even if they have identical relative states (contrary to the single-mind agent of Tappenden [29]). The agents in the sleeping pill experiment [43] can be distinguished in an experiment measuring their position in space. This provides operational meaning of probability for these agents. One may try to assign operational meaning for probability for agents that differ only in their outfits through measurements of the color of the outfit, but this will not be a probability of self-location of the agents in a particular world. It will be a probability for the outfits, not for the agents.

Note that although for various analyses, it is crucial that in the moment we try to define the probability of self-location there is a matter of fact about belonging to a particular world, an agent may nonetheless have other reasons for placing bets on different outcomes. He can do this because he cares more for some of his descendants than others, being aware that all of them will exist (see [43,44]).

## 4. Against a Spacetime Worms View of Agents

Even among proponents of the MWI, the majority are reluctant to accept that we cannot ask a simple question: What is the probability of an outcome of a quantum measurement? It seems that at least after the measurement, when the worlds with different outcomes are created, the question is legitimate. It is very natural that in different worlds live different agents, so apparently, we can ask: In what world do I (the agent) live? This question also fits well with the view of “spacetime worms” [45], according to which an agent is defined as a four-dimensional worm in spacetime. There is a matter of fact about the world in which a particular four-dimensional worm appears. However, I argue that this approach fails. The ontological counterpart of an agent in the MWI is the wave function of the particles from which he is composed. Until the agent becomes entangled with the result of the quantum measurement, there is only one entity like this. In a deterministic theory such as MWI, the entity will evolve into a single particular “thing”. This “thing” can be a set of agents, but we cannot have an uncertainty about what the entity will become.

The lack of operational meaning has not prevented authors from making semantic statements considering two Kirks. Saunders [46], then Wallace [47], then Lewis [48] argue that we can consider two Kirks before the measurement, as can be seen in the quote from Lewis [48]:

We can, if we wish, adopt the Lewis criterion for personal identity in Everettian contexts, in which case there are (in that sense) two persons prior to measurement. We could even give these two persons names, say ${\mathrm{she}}^{↑}$ and ${\mathrm{she}}^{↓}$, so that ${\mathrm{she}}^{↑}$ refers to the person who sees ‘up’ and ${\mathrm{she}}^{↓}$ refers to the person who sees ‘down’. If I walk into the lab and ask “What result is she about to see?”, I might be told “${\mathrm{she}}^{↑}$ will see ‘up’ and ${\mathrm{she}}^{↓}$ will see ‘down’, and at the moment ${\mathrm{she}}^{↑}$ and ${\mathrm{she}}^{↓}$ coincide”.

Saunders and Wallace [35] even claim that we can adopt Lewis’s approach that there are two persons present before branching not only for divergent worlds, but also for branching worlds:

… it is now rather clear, from [35] (Section 2), what we are ignorant of: we do not know which world—which branch, the big bang to the end-of-time—is ours. It is lack of knowledge *de se*, uncertainty of where we are located, not as a stage *S* but as a world-stage 〈W,S〉 or world-time 〈W,t〉, among the branching worlds. Ignorance on this score makes rather obvious sense in the case of diverging worlds, and now we are in a position to see that it makes just as much sense, in our semantics, in the case of branching worlds.

I find that these formal metaphysical attempts lead only to difficulties. The standard physics paradigm relies on agents described locally in spacetime points. The MWI branching structure does not support the diachronic identity of a person toward the future. If ${\mathrm{she}}^{↑}$ and ${\mathrm{she}}^{↓}$ coincide, they cannot lead to different “she’s” later. When ${\mathrm{she}}^{↑}$ and ${\mathrm{she}}^{↓}$ “coincide”, there is nothing to distinguish them. Thus, if we do want to consider “she” as a spacetime worm, we must have a picture of “splitting worms”. “She” before the measurement splits into ${\mathrm{she}}^{↑}$ and ${\mathrm{she}}^{↓}$. This picture still provides a unique description of “she” in the past. Before the measurement, the identity of ${\mathrm{she}}^{↑}$ who sees ‘up’ is “she prior to measurement”. This is also the identity before the measurement of ${\mathrm{she}}^{↓}$ who sees ‘down’ after the measurement. In contrast, the future of a “she” is not another “she”, but rather a set of “she”s.

The proponents of the worm view often draw the analogy with roads with different names, which have nevertheless a partial overlap. A driver, moving in the common part, may ask herself a question about the road she will choose when the roads separate. She might know this before splitting or may decide when she arrives at the fork. However, if she is like a photon reaching a beamsplitter that splits and goes in both ways, then there is no matter of fact before the spitting which path she will take. The worm view with past and future has a perfect sense if we add some ontological entity, like a Bohmian position in configuration space of the agent’s particles (see [1]), or an ontic “mind” of an agent in the Albert–Loewer approach [49]. I see no room for a future evolving worm when the only ontology is the quantum state of the universe. Returning to the analogy with overlapping roads, at the place of the overlap, we do have an additional ontological entity: the name of the road.

## 5. Modified Quantum Teleportation and Albert’s Conclusions

There is no consensus among proponents of the MWI regarding what is a world in the MWI, so it is not surprising that worlds might lead to confusion in understanding self-location of an agent. Undoubtedly, the concept of a centered world is fruitful in many situations, but it has some ambiguities (see [50]). I argue that we can discuss the self-location centering the agent directly.

Due to the locality of physical interactions, we have an operational meaning for self-location of an agent in space. An agent can see what is around him and, comparing with a map, know where he is. So, a modification of Albert’s quantum setup which allows the concept of probability of self-location is teleportation of Kirk to different locations (as it had to be in the classical case). According to the result of the quantum experiment, Kirk is sent to the left location in a green outfit or to the right location in a blue outfit, so we get two Kirks after teleportation. The Kirks with closed eyes can bet on the place they landed on the planet as in the “sleeping pill” setup [43]. The question of self-location (in space) introduces uncertainty even for agents who know everything about the relevant ontology of the situation, e.g., all relevant details of the two Kirks on the planet. Both quantum and classical cases have the peculiar property of subjective uncertainty in a state of complete objective knowledge.

Two referees of this paper expressed similar concerns about the special role of spatial differences which I introduced in the modification of Albert’s setup. I find it helpful to quote one of their comments:

I was puzzled by this particular role that spatial position is supposed to play. If branches have decohered, why can’t other properties play this role of differentiating the two agents? Imagine, for instance, the following scenario: although Kirk is unaware of it, mosquitoes on this planet are attracted by the color blue. Blue Kirk hears a mosquito flying around, while Green Kirk hears nothing. There is clearly a difference in their mental state, so these are two different agents at the same spatial location, but being unaware of the attraction of mosquitoes, they are also unaware of the measurement outcome.

In the semantics of [32] this type of situation corresponds to “tainted self-location uncertainty”. There are two Kirks and they have self-location uncertainty, but it is of the trivial kind, different from uncertainties discussed here and in Albert’s lecture. Contrary to the situation in my modified setup, Kirks are ignorant of an objective relevant fact: the biological property of mosquitoes.

Toward the end of his lecture [27], Albert reaches the conclusion that the claims about probabilities of self-location are “irreducibly indexical”. He argues that

We should not say, and the science of such probabilities should not aspire to say, that this or that theory of the assignment of self-locating probabilities is well confirmed by experiment, or that there are good reasons to believe it, or that it is true or false of the world. What we should say, and all that the science of such probabilities should aspire to say, is that the theory in question is well confirmed for *me* by experiment, and that there are good reasons for *me* to believe it, and that it is true or false of the world that is centered on myself…

Albert considers this conclusion outrageous:

…the important thing to say is that, if probabilities like these are supposed to play a central role in scientific explanations, if probabilities like these are supposed to play the role for example of quantum mechanical chances, then this way of thinking is going to radically diminish the traditional objective realistic aspirations of the scientific project, and the question is why in the world we would even want to fool around with crazy sh*t like this, when there are sensible and workable and flat-footedly mechanical ways of solving the measurement problem on the table…

Yes, the probability of self-location is intrinsically subjective. There is no way for an external agent, a super-technology which can manipulate and measure superposition of Kirks wearing different outfits, to confirm or disconfirm Kirk’s subjective self-location probabilities. However, the agent’s subjective probabilities in the modified Albert’s teleportation to different locations are objective properties of our universe. They are confirmed by *my* subjective evidence as an agent performing quantum experiments. Formally, Albert is correct: In a single world of quantum mechanics with collapse, my empirical evidence is the only one that exists, so it can be named objective instead of subjective. However, since the experience of agents in every Everett world is identical to the experience of an agent in the single world with corresponding collapses, I fail to see a difference in empirical evidence of the agents in the two cases. The standard objection about the definite existence of maverick worlds in MWI, in contrast with only a low probability of existence of a maverick world in the universe with collapses, seems to me a manifestation of the known difficulty of the frequentist approach to probability without infinite ensembles.

I agree that this is a radical change of the traditional way of thinking that a law of evolution of ontic entities (the law of evolution of the quantum state of our universe) is not the full description of nature and we have to complement it by a postulate about the subjective self-location probabilities of sentient agents (the counterpart of the Born rule in MWI). However, I think that this radical change is forced upon us by the many-worlds picture. The quantum theory without collapse also forces us to accept the existence of a macroscopic superposition corresponding to a dead and alive cat, which is viewed as absurd by Schrödinger. And the reason to take MWI seriously is that alternative solutions of the measurement problem have arguably worse features: action at a distance and randomness (see [51]).

## 6. Quantitative Analysis: The Born Rule

I introduced two modifications of Albert’s setups: (1) the agent decides what is the winning option of the bet, and (2) in the quantum case, the position of the agent himself is changed according to the result of the Stern–Gerlach experiment. These changes allow for a legitimate concept of subjective probability in an operational way. It might well be that Albert accepts (1) implicitly; (2) seems to be crucial: without it, Kirk cannot ask the question of self-location.

Now, we can turn to the quantitative analysis. What is the numerical value of the probability for a particular self-location? We have vast empirical data showing that the probability follows the Born rule, but we also have a very extensive literature suggesting that this result can be derived from unitary quantum mechanics alone, e.g., [39,52,53]. I believe that the Born rule cannot be proven without some additional assumptions [8], and that the current proofs either take some implicit assumptions, or use incorrect argumentation based on manipulation of meaningless concepts like the probability of self-location of Kirk in the unmodified Albert’s quantum case.

It is straightforward to show, based on world counting, that the case of agents belonging to equal-amplitude worlds corresponds to the Born rule probability. Saunders [54], using the decoherent histories formalism, argues that agents can always be represented in this way, providing a new route to probability. He continued this approach [55], but questioned the role of decoherence: “Does quantum probability always involve decoherence?” Following the ideas of Boltzmann and Gibbs, Saunders suggested finite quantum frequentism [56] by counting the number of equal-amplitude parts of the wave function corresponding to a particular outcome and dividing it by the total number of such equal-amplitude parts in the wave function. Although this approach provides a nice description of the Born Rule, I do not see in which sense it might be considered as its derivation. It is based on the analogy with classical statistical theory, but there is a difference: in the classical case, always there is a matter of fact (maybe unknown to us) about the state of a system, while in the quantum case (without hidden variables), there is no matter of fact about the part of the quantum wave to which the system belongs, so there is no place for uncertainty.

Another difficulty of Saunders’s approach appears in the analysis of a Stern–Gerlach experiment with a spin in the initial state very close to one of the measured spin-component eigenstates, say “up”. The physics of the detector showing “up” is very much the same as the physics of the detector showing “down”, but since the probability of “up” is much larger, we must have many more orthogonal parts of the wave function of equal amplitude for “up” than for ”down”. Thus, the parts corresponding to “up” must have a very different shape than those corresponding to “down”. This represents tension with the principle of counting all equal-amplitude parts equally. See another analysis of Saunders’s proposal by Khawaja [57].

One can also find authors, e.g., Putnam [58], who treat all agents with non-vanishing amplitude states on an equal footing, even if the amplitudes of the corresponding states are different. In this way, the derived probability rule within MWI contradicts empirical data and leads them to dismiss the MWI. The basis for these derivations is the “naive principle of indifference” (see [59]), according to which all options compatible with the evidence of the agent should be assigned equal probability. The rationale for applying the principle of indifference for self-location beliefs is that, by construction, the agents in all cases have the same sentient state. Moreover, since the agents are placed in separate locations, different unitary operations can be applied in these locations, ending up in a situation in which all agents and their nearby environments will have identical quantum states. (Locality suggests that each local unitary operation cannot change the probability of self-locations. Although in general, locality does not prevent changes in the location of the local operation, the probability of self-location cannot be changed locally because the total probability is 1 and a local change of the probability invariably changes some probabilities in remote locations which locality does not allow.) Thus, we reach a situation in which local agents not only have the same sentient state, but also have no means of distinguishing between different locations by investigating their surroundings, in contrast to Dr. Evil and the brain in a vat discussed in [18]. This type of analysis apparently led Putnam to apply the indifference principle in this case.

However, I argue that this is a mistake. Subjective indistinguishability is not enough. The indifference principle requires that we have no reasons to prefer one location over the other. This is not the case here. We can arrange identical quantum states by local actions in all parameters except for one—the absolute value of the amplitude of the terms in the superposition corresponding to agents in various locations. If the amplitudes are different, then the agents are different, so we cannot claim that “there are no reasons to prefer one over the other”.

A very natural assumption that has roots in the “additivity requirement” of Everett [10] is that the local splitting of an agent into a number of agents in close-by locations should lead to the sum of the probabilities to be found in all of them to be equal to the probability to be found in this place before the splitting. (It is similar to *Weak connection with transformations*, the third “reasonable” axiom of Short [60]). This property follows from locality in a similar way as discussed above regarding the independence of the self-location probability of an agent undergoing local unitary evolution. Locality prevents immediate changes of the probability of self-location in a bounded region. By introducing more splittings (see [52]), we can arrange equal amplitudes for all agents. I [61], suggested adding local operations that make all local states that describe agents identical. Arranging these states in a geometrically symmetrical configuration leads to really identical agents, so there will be no way to prefer one over the other. In this scenario, the principle of indifference can be applied to derive an equal self-location probability of split agents of equal amplitude. This construction, together with the standard quantum formalism, leads to the Born rule for the general case of unequal-amplitude agents. Zurek [62], makes another assumption to derive the Born rule. He assumes a natural “envariance” feature of entangled systems. Wallace [63] puts Deutsch [52] ideas on more rigorous grounds by evoking postulates of decision theory. I find that Wallace’s postulates include some assumptions that go beyond unitary quantum mechanics.

All interpretations of quantum mechanics have the Born rule as an additional postulate. (I am skeptical about Valentini’s attempt to derive the Born rule in the framework of Bohmian mechanics, [64]). In the MWI, the situation is worse: even defining the postulate of the Born rule is difficult because it does not correspond to any objective statement about the ontic state of the universe. The concept of self-location probability in special situations of an agent who is uncertain about the world in which he is (when there is a matter of fact about it) allows us to define the ignorance probability in a familiar betting setting. The postulate that the agent, ignorant of the world in which he is in, has to bet according to the “measure of existence” of this world (see [51]), explains the Born rule statistics for experiments performed in such a way; that is, in experiments with a stage in which the results are obtained, macroscopically different agents are created, but they are still ignorant about the outcome. Most experiments are not like this, so the postulate of betting according to the measure of existence does not lead to a formal derivation of the Born rule in these cases. However, it does not sound plausible that blindfolding or not blindfolding of the observers before quantum experiments affects the statistics of the results of their experiments, in particular, because it can lead to superluminal signaling. Note that Tappenden [11] argued more than a decade ago that just a possibility of blindfolding of the observer, which gives him ignorance of the probability of self-location, allows defining the Born rule postulate in the MWI.

Maybe the simplest way to make a postulate is to declare that we should expect to be located in the world with the Born rule statistics of the results of quantum experiments. Clearly, this postulate does not provide a very informative explanation of the Born rule; however, I find it meaningful. It is a statement about our subjective information in a particular world, but it describes the law of nature relevant to the whole universe of the MWI. It is an unusual law. It is about subjective experiences of sentient creatures in the universe. This law does not follow from the unitary dynamics of quantum theory. Indeed, unitary evolution is consistent with other self-location probability postulates including the naive law: the probabilities of self-location in every world with a nonvanishing measure of existence are equal. This law was viewed by Putnam as the most natural one, but of course, it contradicts our empirical evidence. Postulating the Born rule does not nullify the value of its numerous derivations based on various natural assumptions because these derivations show the plausibility of the postulate.

## 7. Conclusions

The concept of self-location probability leads to debates that span from speculation that we live in a computer simulation, to arguments that the MWI is inconsistent, and to a controversy about the proof of the Born rule. Adlam [15] just published a paper “Against self-location”, while Chen [65] writes:

As the case study shows, postulating self-locating probability in physics is like opening a Pandora’s box: it is full of conceptual difficulties. We may wonder whether it is appropriate to allow self-locating postulates in physics

I believe that these difficulties appear when we use an abstract approach to science considering a wide range of metaphysical options. I argue that if we limit ourselves to standard practice in physics grounded in operational meaning, this concept is useful and even necessary. The confusion, controversy, and paradox of the probability of self-location follow from the formal concept of “self”. Considering “self” as an entity which is local in space, local in time, and which is macroscopically different from any other self (possibly only due to location in space) allows a satisfactory concept of probability of self-location which keeps the nontrivial feature of having subjective ignorance of self-location without ignorance about the state of the universe.

## Data Availability

No new data were created or analyzed in this study. Data sharing is not applicable to this article.
